# Efficient Spin-Selective Electron Transport at Low Voltages of Thia-Bridged Triarylamine Hetero[4]helicenes Chemisorbed Monolayer

**DOI:** 10.1021/acsnano.3c04878

**Published:** 2023-07-26

**Authors:** Niccolò Giaconi, Lorenzo Poggini, Michela Lupi, Matteo Briganti, Anil Kumar, Tapan K. Das, Andrea L. Sorrentino, Caterina Viglianisi, Stefano Menichetti, Ron Naaman, Roberta Sessoli, Matteo Mannini

**Affiliations:** † Department of Chemistry “Ugo Schiff” (DICUS) & INSTM Research Unit, University of Florence, Via della Lastruccia 3-13, Sesto Fiorentino 50019, Italy; ‡ Istituto di Chimica dei Composti Organo-Metallici (ICCOM-CNR), Via Madonna del Piano 10, Sesto Fiorentino 50019, Italy; § Department of Chemical and Biological Physics, Weizmann Institute of Science, Rehovot 76100, Israel

**Keywords:** Chirality, Helicenes, Self-assembled monolayers, molecular spintronics, CISS effect

## Abstract

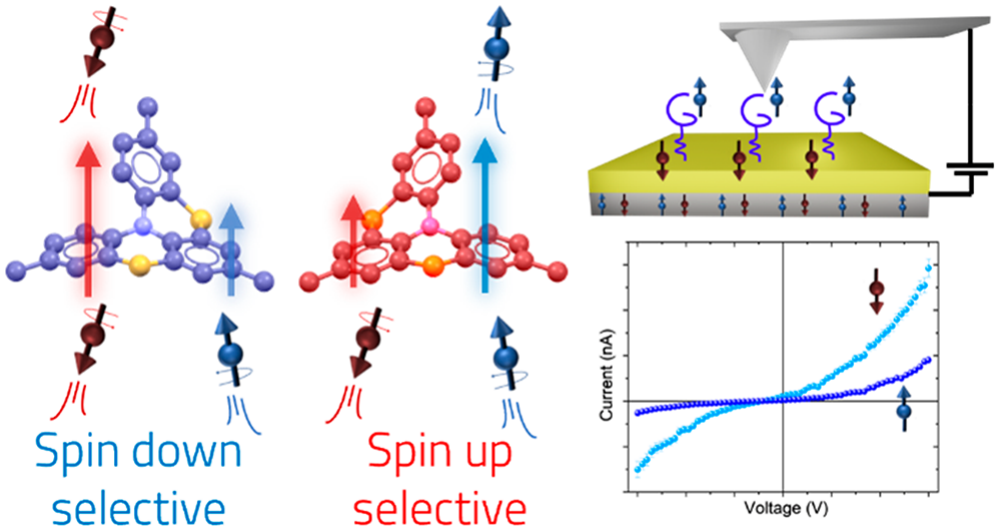

The Chirality Induced Spin Selectivity (CISS) effect describes the capability of chiral molecules to act as spin filters discriminating flowing electrons according to their spin state. Within molecular spintronics, efforts are focused on developing chiral-molecule-based technologies to control the injection and coherence of spin-polarized currents. Herein, for this purpose, we study spin selectivity properties of a monolayer of a thioalkyl derivative of a thia-bridged triarylamine hetero[4]helicene chemisorbed on a gold surface. A stacked device assembled by embedding a monolayer of these molecules between ferromagnetic and diamagnetic electrodes exhibits asymmetric magnetoresistance with inversion of the signal according to the handedness of molecules, in line with the presence of the CISS effect. In addition, magnetically conductive atomic force microscopy reveals efficient electron spin filtering even at unusually low potentials. Our results demonstrate that thia[4]heterohelicenes represent key candidates for the development of chiral spintronic devices.

One of the major challenges in the field of molecular spintronics^1^ is understanding the mechanism of injection and transport of spin-polarized currents from/to a molecular layer. This is a crucial step for improving the performances of molecular-based spintronics devices and their use in data storage and quantum computing.^2^ Due to their advantageous properties, such as low spin–orbit and hyperfine couplings, organic molecules have been considered optimal units for developing spintronic devices.^3^ Indeed, these coupling effects, which occur mainly in inorganic materials, are considered detrimental to preserving spin coherence over time and distances. Molecular systems have been used to modify the spin interface, strongly influencing transport and magneto-transport processes.^4−7^ Commonly, both organic materials and organic molecules have been exploited as a medium to promote the transport of spin-polarized currents.^8^ Recently, the use of structural characteristics of molecules to influence the spin polarization of the flowing current has emerged. In particular, the conduction through chiral molecules can be spin-selective,^9^ promoting electron transport in a specific spin state. This phenomenon, discovered by Naaman et al.^10^ and renamed Chirality Induced Spin Selectivity (CISS) effect,^11^ suggests the use of a chiral molecule as a spin-selective agent.^12^ CISS has been observed for several molecules of biological interest, such as oligopeptides,^13,14^ DNA,^15^ and proteins,^16^ and also for molecules like helicenes.^17,18^ The spin selectivity properties of the latter have been also theoretically modeled considering the key role of spin–orbit interactions^19^ and make these molecules suitable as a tool for quantum technologies working at room temperature.^20^ Recently, some of us studied and developed a deposition procedure of a thia[4]helicene radical cation^21^ on a surface at the submonolayer coverage exploiting noncovalent interactions between those molecules and a thiophenol templated Au(111) substrate.^22^ Herein, moving toward a more robust architecture, we investigated the CISS effect on a neutral thioacetyl derivative of thia[4]helicene that was synthesized and chemically anchored on a gold surface for this purpose. The spin-selective transport properties of the molecular deposit were studied by assembling a molecular-based spintronic device, *i.e.*, vertical spin valve, embedding a molecular monolayer and measuring the magnetoresistance varying magnetic field and temperature. The inversion of the signal according to the handedness of the helicene was observed, confirming the active role played by molecules in the spin filtering process. Besides, magnetic conductive-atomic force microscopy (mc-AFM) was employed to measure the current flowing between the tip and the sample under a magnetic field. A high percentage of spin polarization at room temperature was also observed working at a low applied potential.

## Results and Discussion

**Scheme 1:**
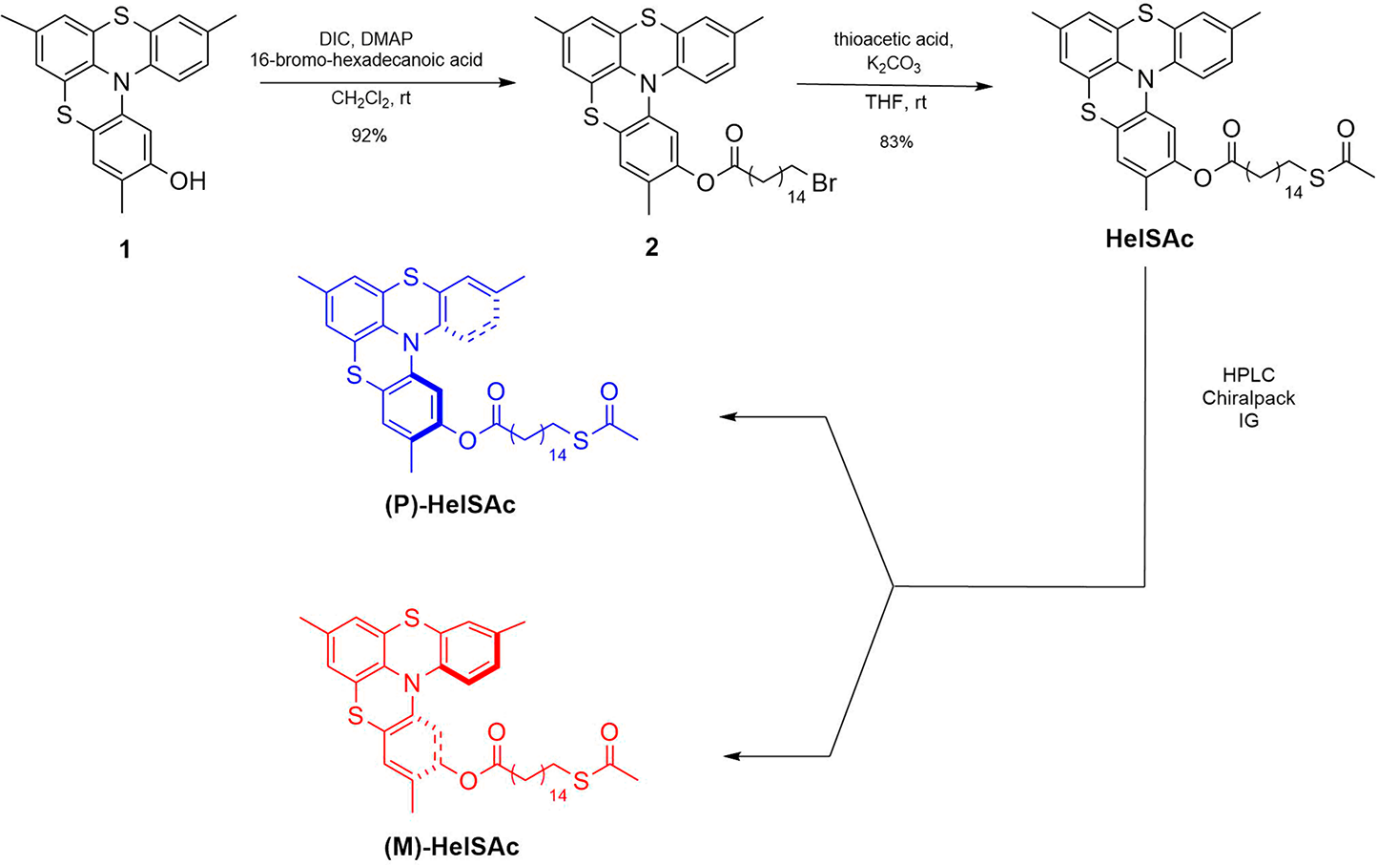
Synthetic Route to Obtain **(***P***)-HelSAc** and **(***M***)-HelSAc** Compounds

The synthetic route to obtain enantiopure thioacetyl derivatives is outlined in Scheme 1. Hydroxy-substituted thiahelicene (**1**), obtained according to the procedure already reported in literature,^23^ was reacted with 16-bromo-hexadecanoic acid in the presence of diisopropylcarbodiimide (DIC) and *N*,*N*-dimethylamino pyridine (DMAP) in dry CH_2_Cl_2_ to give ester **2** isolated in 92% yield. The resulting compound (**2**) was then reacted with thioacetic acid and K_2_CO_3_ in dry tetrahydrofuran (THF) affording the thioester (**HelSAc**) in 83% yield. Enantiopure derivatives were obtained through semipreparative high-performance liquid chromatography (HPLC) on a chiral stationary phase affording the right-handed enantiomer **(***P***)-HelSAc** (first eluted, [α]_D_^25^ + 180°; *c* = 5 × 10^–3^, CH_2_Cl_2_) and the left-handed one **(***M***)-HelSAc** (second eluted, [α]_D_^25^ – 180°; *c* = 5 × 10^–3^, CH_2_Cl_2_) both with enantiomeric excess (e.e.) ≥99%. The absolute configuration of the single enantiomers of derivatives **HelSAc** was assigned as (*P*)-(+) and (*M*)-(−) by comparison with our previous results on helicenes of type **HelSAc**.^24−26^ Additional details about the synthesis can be found in the Supporting Information (Figure S1 to Figure S4).

**Figure 1:**
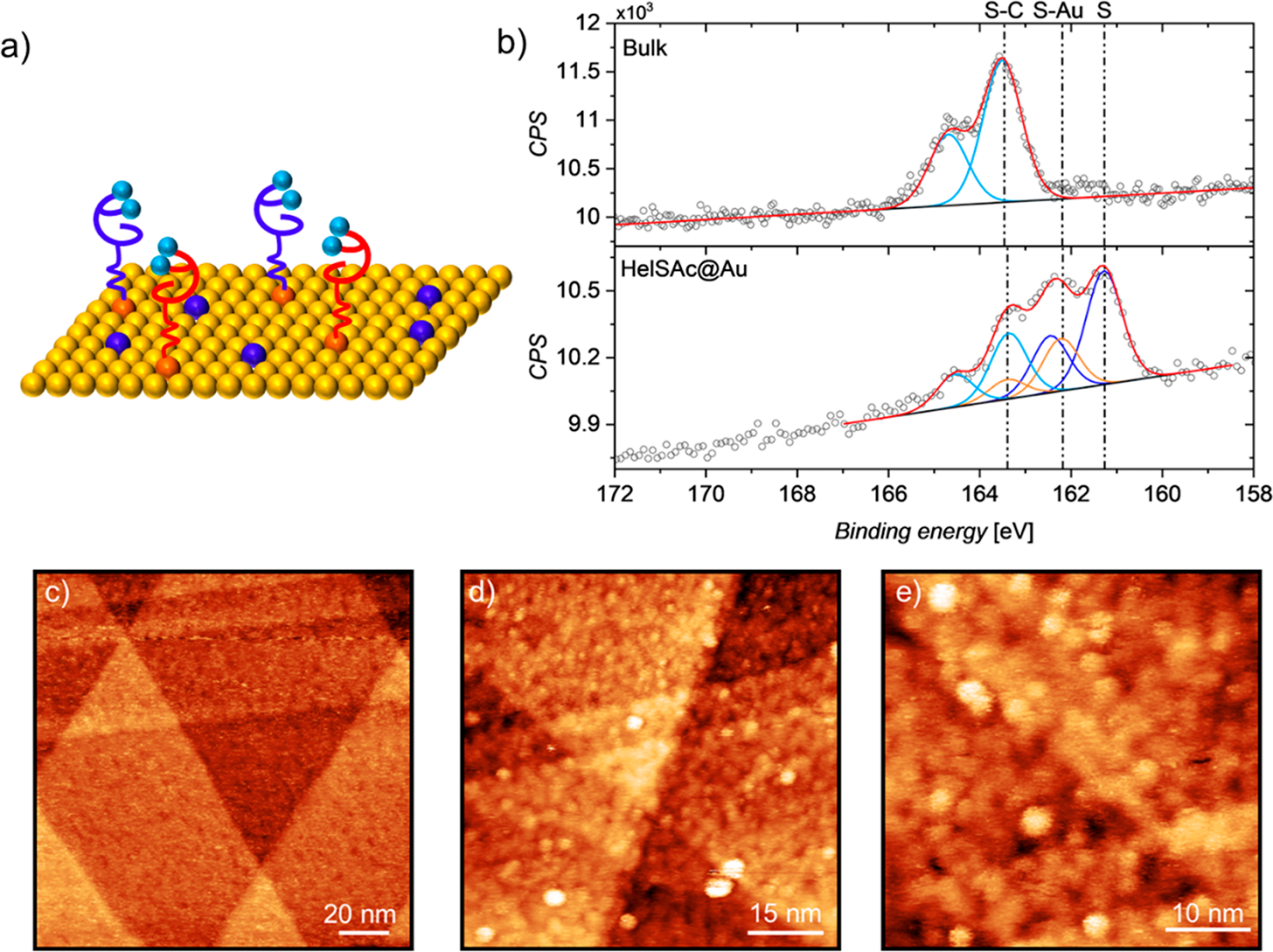
a) Scheme of deposition of **HelSAc**. Spheres represent sulfur atoms in a different chemical environment: bound to the Au surface (orange), in the helicene backbone (cyan), and atomic sulfur (blue). b) S2p regions of **HelSAc** bulk sample (top) and of **HelSAc@Au** monolayer (bottom). Colors of the best fitting components reflect the previous description. Each signal is accompanied by its spin orbit coupled S2p_1/2_ component shifted by 1.2 eV. STM images of **HelSAc** monolayer assembled on Au(111) surface recorded at 30 K. c) 150 × 150 nm^2^, *V* = 0.1 V, *I*_*t*_ = 300 pA; d) 75 × 75 nm^2^, *V* = 1.2 V, *I*_*t*_ = 2 pA; e) 45 × 45 nm^2^, *V* = 1.2 V, *I*_*t*_ = 2 pA.

Aiming to investigate spin filtering properties, a monolayer of **HelSAc** was assembled on a gold surface exploiting the spontaneous deprotection of thioacetyl functionalization of a 2 mM solution in dichloromethane at room temperature^27^ under the presence of gold. The deposition was performed by a *wet-chemistry* approach, thus achieving a self-assembled monolayer (SAM, **HelSAc@Au**, Figure 1a). At the end of the incubation, several cleaning cycles with pure solvent were performed to ensure the removal of excess molecules left physisorbed on the surface.

The deposition of **HelSAc** was studied by X-ray photoelectron spectroscopy (XPS). N1s, C1s, and S2p regions were investigated to characterize the sample and compare the results with those obtained from a reference bulk sample of **HelSAc***via* dropcasting from dichloromethane. N1s XPS spectra of bulk **HelSAc** (Figure S5) feature one component at 399.8 eV, corresponding to the only nitrogen chemical environment present in the molecule.^28^ This component is also present in the N1s XPS spectra of **HelSAc@Au** (at 399.9 eV). The analysis of the C1s region of both bulk and **HelSAc@Au** samples (Figure S6) suggests the presence of one strong component at 284.3 eV attributed to C–C/C=C species^29^ and another one at 285.4 eV assigned to C–S/C-N.^30^ In addition, spectra feature two minor components at higher binding energy due to the presence of C–O/C=O functional groups that might come from environmental contamination.^31^

The most significant information about the assembly of **HelSAc** on the surface can be extracted by analyzing the S2p region. **HelSAc** bulk sample (Figure 1b) features a single major component, and its spin–orbit related one, at 163.4 eV; this is attributable to sulfur atoms in the helicene backbone and thioacetyl group that cannot be distinguished. In the S2p region of the monolayer, a significant change in the line shape of the spectra is noticed due to processes occurring during the deposition procedure. Indeed, three components are needed to reproduce the experimental data. The component already detected in the bulk sample at 163.4 eV was given by sulfur atoms in the helicene structure plus a new component at 162.2 eV arising from the formation of covalent bonds between gold substrate and deprotected sulfur atoms of thioacetyl groups. The formation of a chemisorbed layer of intact molecules on surfaces is further corroborated by the ratio between these two components, which is comparable with the theoretical one (2:1). An additional component is present at lower binding energy (*ca*. 161.3 eV) due to partial cleavage of carbon–sulfur bonds that leaves atomic sulfur adsorbed on the gold surface, as already observed in literature.^32−34^ The presence of these defects (like others typically present in all self-assembled monolayers)^35^ in the monolayer formation is not expected to significantly affect the CISS experiments that will be described hereafter. A semiquantitative evaluation of the elemental composition of both bulk and monolayer samples was obtained (see Table S1). In the limit of the experimental error of the XPS and excluding the contribution of atomic sulfur, the measured percentages are in good agreement with the theoretically expected stoichiometry.

Scanning tunneling microscopy (STM) images were recorded to evaluate the morphology of the molecular monolayer on the metallic substrate. The annealed bare gold surface reported in Figure S7a is characterized by atomic terraces, and a periodic herringbone reconstruction pattern can be appreciated resulting from the spontaneous formation of “stress domains”.^36^ In Figure 1c we report a 150 × 150 nm^2^ image of the molecular monolayer assembled on the annealed surface. The gold terraces are still visible, thus indicating homogeneous growth of the molecular deposit. In addition, pinholes are present as expected when the thioacetyl-protected thiols self-assemble on gold with the spontaneous removal of the protecting group.^37,38^ Increasing the magnification of the scanned area and operating with different tunneling conditions (Figure 1d,e), it is possible to appreciate the presence of disordered dots whose lateral dimensions might be consistent with those of lying molecules, according to theoretical calculations described in detail further in the text. A statistical analysis was performed on Figure 1e extracting, as the peak of a log-normal distribution, an average value for the diameter equal to 1.8 nm (Figure S7b) and estimating a density packing of about 0.6 molecule/nm^2^.

**Figure 2:**
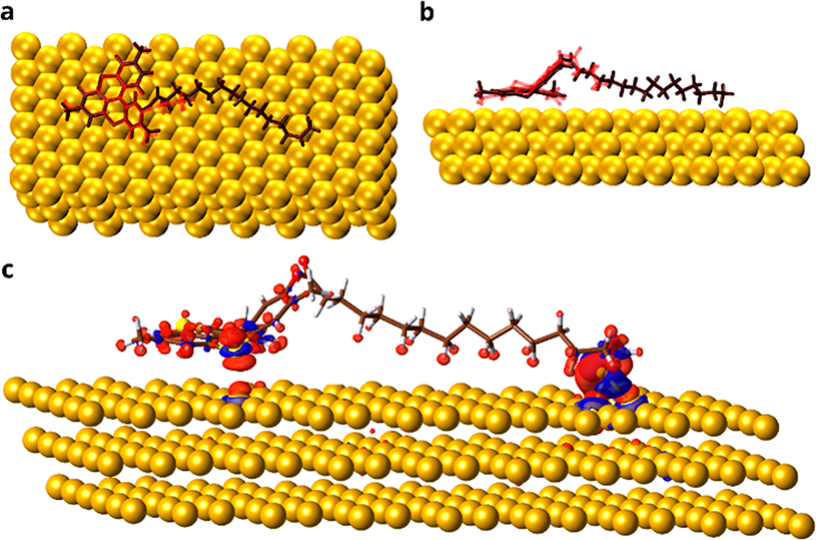
Optimized structure of **(***P***)-HelSAc** on Au(111) calculated by DFT. a, b) Side and top views of the adsorbed structure (brown) overlaying the optimized structure in the vacuum (red). c) Computed electron density difference. Blue and red isosurfaces (drawn for a value of 0.0014 e bohr^–3^) correspond to reduced and increased electron densities, respectively, compared to the non-interacting component (isolated **(***P***)-HelSAc** and Au surface). C, N, S, O, H, and Au are brown, blue, yellow, red, white, and dark yellow, respectively.

**Figure 3:**
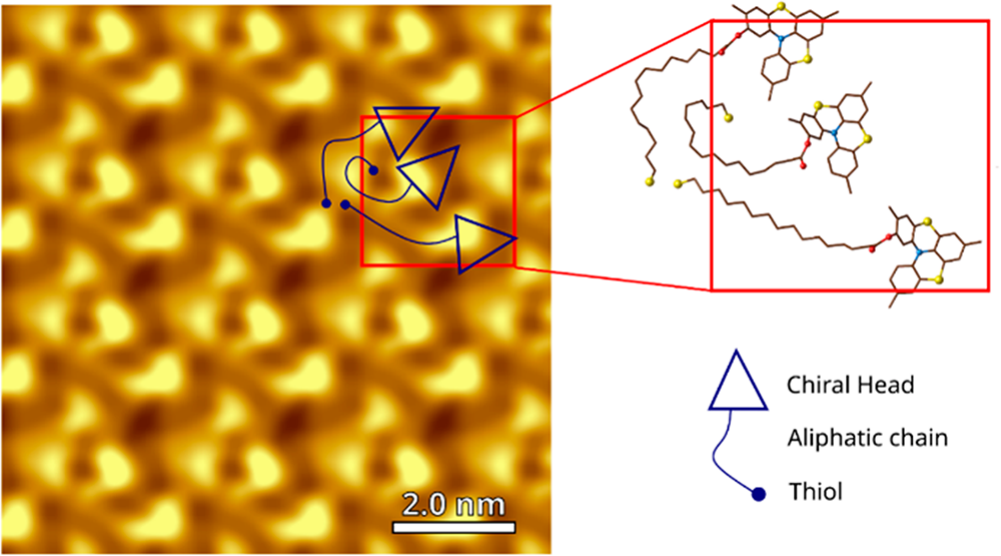
pDFT simulated 9 × 9 nm STM image (*V* = +2 V) of a monolayer of **(***P***)-HelSAc** on Au(111), with superimposed optimized geometric structure. The red rectangles indicate the simulation cell. C, N, S, and O are brown, blue, yellow, and red, respectively. The gold surface is not shown for the sake of clarity.

Periodic density functional theory (pDFT) simulations were performed to gain further insights into the adsorption process. First, a single unit of **(***P***)-HelSAc** was optimized on a clean Au(111) surface. As already observed^39,40^ the S–C bond undergoes a homolytic cleavage upon adsorption. Consequently, to model the system, the protecting thioacetyl group was removed. Thereafter, the sulfur atom strongly bonds to the gold surface, bridging two neighboring gold atoms. This observation is in agreement with literature reports indicating the bridge sites as the preferred adsorption sites for thiols on a clean Au surface.^41−43^ The average Au–S bond length is 2.5 Å, while the vertical distance from the surface is 2.0 Å, indicating that chemisorption of the thiol group has occurred. However, a computed total adsorption energy of **HelSAc** on Au(111) is 115 kcal/mol: since the adsorption energy of simple alkanethiols on gold is 20–40 kcal/mol, this implies that the thiahelicene head also strongly interacts with the surface (Figure 2a). Although the chirality of the structure is preserved, the thiahelicene part in contact with the substrate is slightly more planar with respect to the isolated geometry. As shown in Figure 2b, the dihedral angle between the aromatic ring in contact with the surface and the plane containing S_thioether_ and N atoms goes from 31.7° to 8.9°, indicating that a strong interaction takes place upon grafting. This is due not only to van der Waals (VdW) interactions among the gold and the conjugated system but also to the presence of the two thioether groups inside the chiral backbone. Indeed, the vertical distance from the surface of one of the two sulfur atoms is only 2.9 Å, well below the sum of the VdW radii of S and Au, 3.9 Å.^44^ This behavior is evident from Figure 2c, which shows an increase in electron density along the S_thioether_-Au interatomic direction. However, due to the rigidity of the helicene structure, only one of the sulfurs can efficiently interact with the surface. As a result of all of these effects, the whole helicene group donates electron density to the surface. This electron transfer is estimated to be of 0.6 e^–^ and 0.3 e^–^, as evaluated by the Mulliken and Hirshfeld population analysis, respectively. Despite their limitations, both approaches describe a significant amount of charge transfer from the chiral group upon adsorption. Although this magnitude of the computed interaction should be considered as an upper limit since an ideal Au surface is considered, which is not always the case when molecules are deposited from solution, all of these findings indicate that a strong interaction of the molecule with the substrate can take place. Then, based on the optimization of the single molecule, to take into account packing effects and intermolecular interactions the SAM arrangement was tentatively simulated by pDFT using three **(***M***)-HelSAc** molecules to mimic a molecular density in agreement with the experimental values extracted from STM. That choice was imposed by the huge number of involved atoms to be handled in this optimization of a gold surface with dimensions 2.5 × 2.6 nm. Even in this case, where for each molecule one sulfur atom of thioether groups is computed 3.0 Å above the surface, we observe the strong interaction with the surface computed for the single molecule. The STM images for the optimized structure of the monolayer were also computed (see Figure 3). The image at +2 V bias shows a complex arrangement of bright spots with irregular shapes. Each corresponds to one of the two benzene rings of the thiahelicene structure. Some clearer spots also arise from the folding of the alkane chain connecting the chiral head to the thiol group. Although the experimental STM images were collected on an SAM made of a racemic mixture of **HelSAc**, the distances among the brightest spots in the simulated morphology vary from 1.2 to 2 nm, in agreement with the experimental STM images.

**Figure 4:**
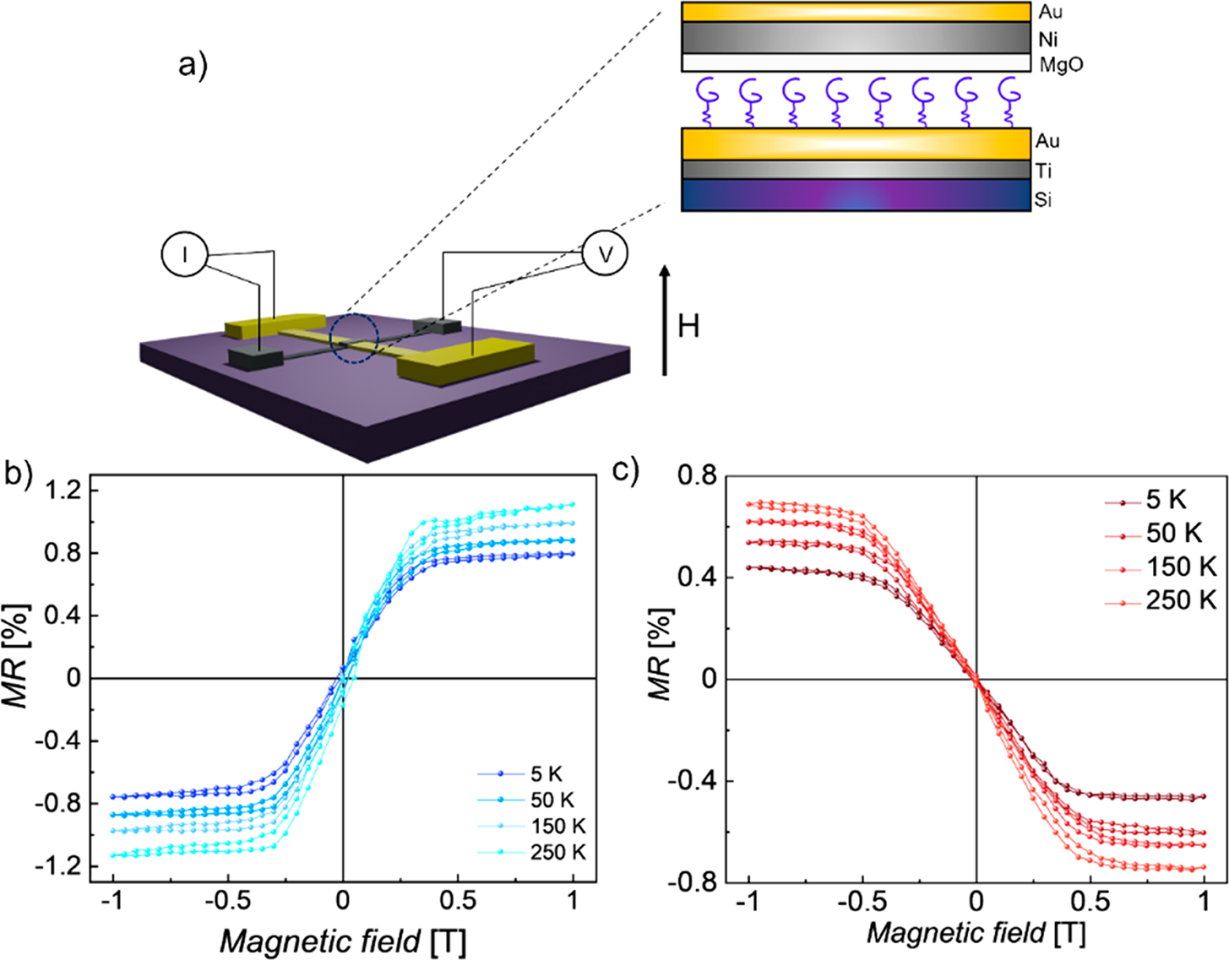
a) Scheme of four-probe MR device setup with details on the cross junction. Temperature-dependent magnetoresistance percentage as a function of the applied magnetic field when a layer of enantiopure b) **(***P***)-HelSAc** or c) **(***M***)-HelSAc** is embedded in the device.

Spin filtering properties of **HelSAc** were initially investigated at variable temperatures by assembling a spin-valve-like vertical device in which one of the two ferromagnetic electrodes is replaced by the chiral molecule assembled on a diamagnetic electrode to perform magnetoresistance (MR) measurements. A scheme of the device is shown in Figure 4a. A self-assembled monolayer of chiral molecules was deposited on a gold bottom electrode, and then 2 nm of MgO insulating buffer layer was deposited on top of the helicene layer, followed by Ni and Au (see Methods section for further details about device fabrication). The current flowing through the molecular monolayer was studied by varying the magnetic field (±1 T) applied perpendicularly to the surface plane in a standard four-probe setup under a constant current of 0.5 mA. Figure 4b,c reports the MR percentage as a function of the applied magnetic field for **(***P***)-HelSAc** and **(***M***)-HelSAc**. The MR (%) was defined as , where *R*_B_ is the resistance measured when an external magnetic field *B* is applied perpendicular to the device surface and *R*_0_ is the resistance measured at zero magnetic field. An asymmetric trend of MR is clearly detected, and the signal inverts its sign upon changing the handedness of the employed enantiomer. The percentage of spin polarization is only around 1%. This small value might be due to the MR measurements in which all the electrons flowing through the device are collected, namely, electrons flowing through the molecular layer and those ejected through pin holes or bare gold. However, only the former are spin-filtered. Temperature dependence of the MR was also investigated. Analogously to what was already observed in other chiral systems, the percentage of spin polarization increases with the temperature.^45−47^ Although a theoretical model that quantitatively reproduces these experimental results is still lacking, it has recently been hypothesized that the role of phonon-enhanced spin orbit-coupling could play a crucial role.^48−51^ Furthermore, it has been demonstrated that molecular vibrations give rise to molecular charge redistribution that could promote spin polarization when chiral molecules are coupled to a nonmagnetic metal.^52^

**Figure 5:**
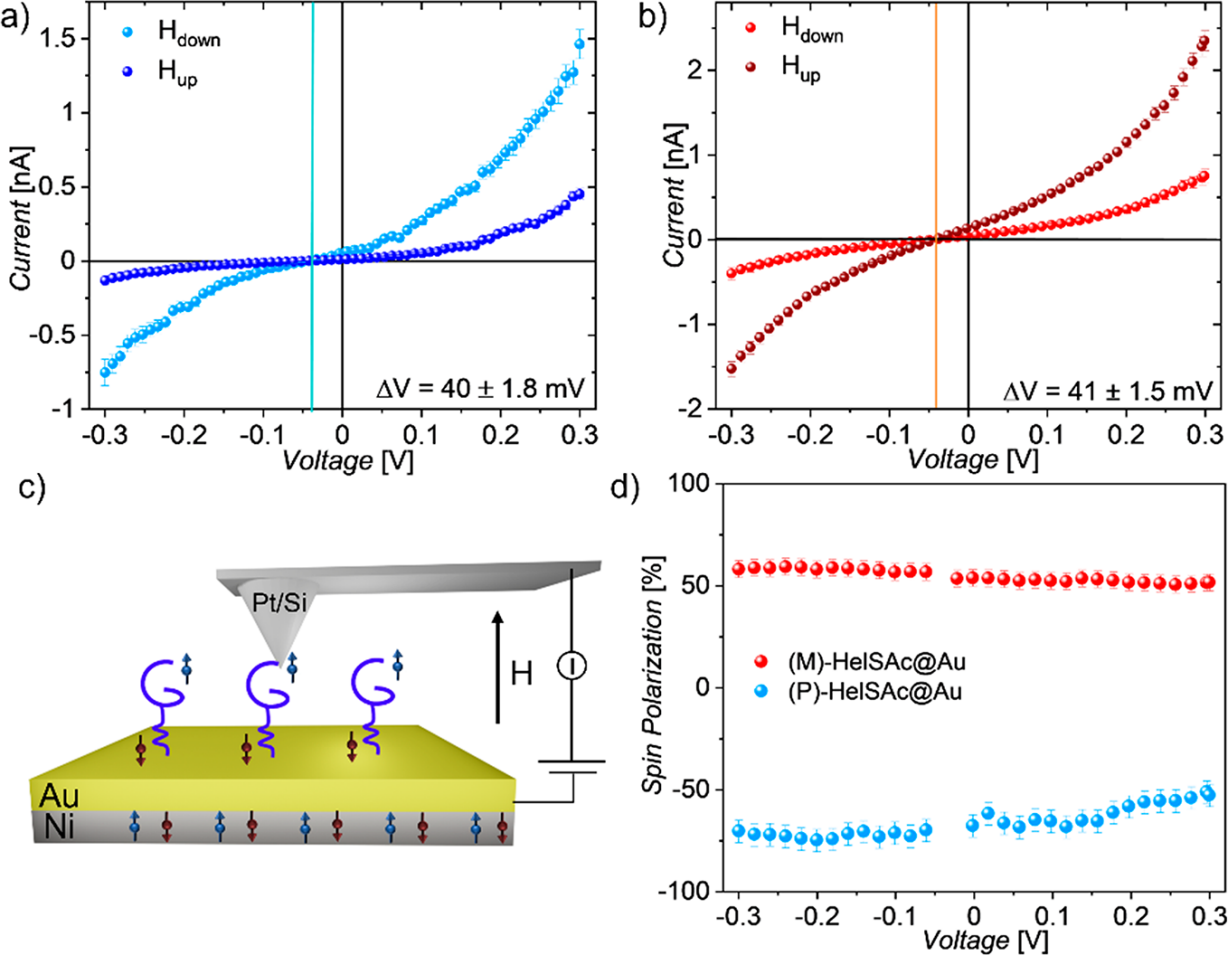
*I–V* curves acquired at room temperature on a) **(***P***)-HelSAc** and b) **(***M***)-HelSAc** over −0.3 to 0–3 V potentiometric window applying either a negative or positive ±0.5 T magnetic field. Vertical lines indicate the zero current point. c) Experimental setup of mc-AFM. d) Corresponding spin polarization percentage extracted from a) and b) *I–V* curves when **(***M***)-HelSAc** and **(***P***)-HelSAc** enantiopure monolayers are used.

A parallel test to evaluate the spin filtering behavior of the helicene monolayer was conducted by performing mc-AFM experiments. This well-known method is proven excellent for local spin-dependent conductivity measurements through molecular layers assembled on a surface.^53^ Molecules were assembled on a silicon wafer with on top a bilayer constituted by Ni/Au (100 and 8 nm thickness, respectively). Measurements were obtained using a Pt-coated diamagnetic and conductive AFM tip (curvature radius <40 nm) under an applied positive or negative magnetic field (±0.5 T) perpendicular to the surface. The bottom layer of Ni promotes the initial spin polarization of electrons injected into the chiral layer. Depending on the direction of the external magnetic field, the spins of Ni substrate can be aligned parallel or antiparallel to the direction of the flowing current. Besides, an electrical potential was applied so that the substrate is biased relative to the tip, which is at ground (the scheme of the experimental setup is reported in Figure 5). The current flowing through the molecular monolayer was measured by keeping the tip in contact with the sample. Data shown in Figure 5a,b result from the average of hundreds of *I–V* curves acquired on the monolayer obtained with the two enantiomers (for further details about corresponding statistical data and monolayer thickness, see Method section and Figures S8 and S9).

The pronounced difference in the current when the field is reversed clearly indicates the occurrence of spin filtering, while the reverse trend for enantiomers with opposite chirality clearly points to a CISS effect induced by the molecular layer. Indeed, when the **(***P***)-HelSAc** monolayer is used, a higher current is registered with negative magnetic fields, namely, with the magnetization of the magnetic layer pointing away from the molecular monolayer, while the opposite behavior is observed with **(***M***)-HelSAc** where a higher value is recorded applying positive magnetic fields (magnetization pointing toward the chemisorbed molecules). Although the *I/V* curves are asymmetric, the same field dependence is observed for opposite bias despite electrons traveling in opposite directions with respect to the chirality vector of the molecules. This is indeed expected for CISS because the spin of the transmitted electrons is also reversed if electrons are injected in the Ni minority spin conduction band, compared to extracting them from the majority spin one. Typically the threshold voltage for injecting spins into chiral molecules is on the order of thousands of mV.^45,54,55^ Here, at variance, it is worth highlighting that the spin filtering process in this system also occurs for very weak applied voltages. Indeed, contrary to previous studies reported in literature^45,46,53,56^ involving DNA, porphyrins, or helicenes, where the CISS effect was detected above ±1.5–2 V, here it is possible to appreciate a relevant difference in current conduction inside a ±0.3 V range. This allows high spin polarization at very low potentials. As shown before with the *I/V* curves, the CISS effect is observed in a nonlinear regime of current dependence on the applied voltage. As a result, in most cases, spin polarization is observed at potentials exceeding 1 V. Here, the spin selectivity occurs already at potentials close to zero, and therefore, the molecules studied can be operated at low voltages, hence low-power spintronics devices. Operations in this low-voltage regime ensure robustness of the molecular devices. Moreover, a zero-shift voltage observed in the *I–V* curves is observed. It might be ascribable to the surface potential that arose due to the assembly of molecular deposits featuring an electric dipole moment on the surface,^57^ confirming the molecular nature of the junction. The spin polarization percentage for both enantiomers was calculated using the following equation , where *I*_up_ and *I*_down_ are the intensities of the current measured applying positive and negative magnetic fields, respectively. As shown in Figure 5d both enantiomers exhibit a high spin polarization percentage at 0.5 T of about 60% at room temperature. From a qualitative point of view, these results are consistent with those obtained with MR characterization. However, the mc-AFM setup, when averaging on a sensible number of measurements acquired avoiding portions of the surface featuring low-quality molecular deposit, yields a significantly higher spin-polarized current compared to the vertical spintronic device because here the measurements are less affected by defects, percolation issues, and short-circuits formed during deposition of the top electrode. The polarization degree at room temperature is higher than reported in the literature^17,56^ for self-assembled monolayers of several chiral molecules and, specifically, for helicene compounds which show spin selectivity lower than 45% once assembled on metallic substrates^18^ as well as when deposited as physisorbed layers on highly oriented pyrolytic graphite (HOPG).^17,59^ Furthermore, it is important to underline that the latter systems feature chirality extended to the whole supramolecular structure, and several studies demonstrated that the magnitude of the spin polarization is proportional to the length of the chiral filter.^13,15,46^ In our case, instead, the achiral alkyl chain does not play any role in the spin-filtering process, and chirality is restricted to a helicoidal system of only four rings with a rotatory optical power estimated to be [α]_D_^25^ ± 180° and, therefore, lower compared to that of other heterohelicenes as well as heptahelicenes studied for CISS applications.^17,18^ Moreover, the helical pitch is estimated to be even diminished by the surface interaction. The spin selectivity is higher than previously detected for a physisorbed monolayer of heterohelicene of similar size but not containing sulfur atoms in the chiral moiety.^58^ In addition, no sizable conductivity and CISS effect were reported below 0.1 V.^17^ Instead, in our case, the more diffuse electron density of the sulfur atoms in the thiahelicene structure can also increase the conductivity by promoting a stronger interaction with the gold substrate.

## Conclusions

In this work, we demonstrated the high efficiency of a monolayer of thia[4]heterohelicenes chemically anchored on surface as spin filtering agent. We deposited an enantiopure self-assembled monolayer of thioacetyl-functionalized molecules on gold. We thoroughly investigated the molecular deposits by performing XPS and STM characterizations of the molecular monolayer; the STM images were flanked by pDFT simulations to gain further knowledge of the molecular deposit. Then we assembled a chiral molecule-based micrometric vertical device for magnetoresistance measurements, demonstrating the successful embedding of these chiral molecules in a real device to control the spin injection process. Finally, performing mc-AFM measurements, we highlighted the excellent local spin filtering behavior of this system at the nanoscale obtaining a high value of spin polarization percentage (above 60%) at room temperature. Although spin selectivity was previously observed on helicenes,^17^ the CISS effect is here detected for surprisingly low bias voltages. Furthermore, to the best of our knowledge, the detected spin polarization is the highest induced by a monolayer of molecules covalently bound to a metallic surface. Even if further investigations are necessary to clarify the role played by the sulfur atoms in determining the high conductivity and pronounced spin filtering of this molecule, this study already highlights the potential of the class of compounds of thia[4]heterohelicenes as possible candidates for the development of chiral spintronic devices. Besides, the preparation of the corresponding radical cation^22^ by performing chemical oxidation may allow to merge chiral structure and paramagnetic properties in a single compound introducing an additional parameter capable of influencing the spin polarization of electrons. Our efforts are focused in this direction.

## Methods

### Synthesis

Details about synthetic procedures of **HelSAc** are reported in the Supporting Information. ^1^H and ^13^C NMR spectra were recorded with a Varian Mercury Plus 400, using CDCl_3_ and CD_2_Cl_2_ as solvents. Residual CHCl_3_ at δ = 7.26 ppm and residual CHDCl_2_ at δ = 5.32 ppm were used as the reference for ^1^H NMR spectra. Central lines of CDCl_3_ at δ = 77.00 ppm and CD_2_Cl_2_ at δ = 54.00 were used as the reference for ^13^C NMR spectra. Fourier transform infrared (FT-IR) spectra were recorded with Spectrum Two FT-IR Spectrometer. Electrospray ionization mass spectrometry (ESI-MS) spectra were recorded with an *J*EOL MStation *J*MS700. Melting points were measured with Stuart SMP50 Automatic Melting Point Apparatus. All the reactions were monitored, and *R*_f_ was calculated by thin-layer chromatography (TLC) on commercially available precoated plates (silica gel 60 F 254) visualizing the products with acidic vanillin solution. Silica gel 60 (230–400 mesh) was used for column chromatography. Dry solvents were obtained by The PureSolv Micro Solvent Purification System unless otherwise specified. Optical rotation measurements were performed on a JASCO DIP-370 polarimeter (JASCO, Easton, MD, USA), and the specific rotation of compounds was reported as follows: [α]_λ_ (*c* (g/mL), solvent). UV spectra were obtained on a Varian Cary 50 UV–vis spectrophotometer. Elemental analysis was measured with a Thermoscientific FlashSmart Elemental Analyzer CHNS/O.

### Enantiomeric Resolution

The HPLC resolution was performed on an HPLC Waters Alliance 2695 equipped with a 200 μL loop injector and a UV Waters PDA 2996 spectrophotometer using HPLC grade solvents purchased from Merck. The semipreparative resolution was carried out on a CHIRALPAK IG semipreparative column (250 × 10 mm/5 μm) purchased from Chiral Technologies Europe. The mobile phase, delivered at a flow rate 3.5 mL/min, was hexane/CH_2_Cl_2_ 70/30 v/v. Enantiomeric excess was measured on a CHIRALPAK IA analytical column (250 × 4.6 mm/5 μm) purchased from Chiral Technologies Europe. The mobile phase, delivered at a flow rate 1.2 mL/min, was hexane/CH_2_Cl_2_ 70/30 v/v.

### Substrate Preparation for XPS and STM Experiments

The substrate was prepared by evaporating gold on a mica substrate inside a vacuum chamber (≈10^–6^ mbar) with a deposition rate lower than 0.1 Å/s. The surface was prepared in ultrahigh vacuum (UHV) by sputtering cycles with Ar^+^ ions and subsequent annealing at 430 K to induce the reconstruction of the Au(111) surface before proceeding with the deposition of molecules. Surface cleanliness and reconstruction were controlled by XPS and STM measurements after preparation.

### XPS Measurements

XPS measurements were performed using a microfocused monochromatic Al Kα radiation source (1486.6 eV, model SPECS XR-MS Focus 600) and a multichannel detector electron analyzer (model SPECS Phoibos 150 1DLD) with a pass energy of 40 eV to ensure an appropriate resolution. Spectra were acquired in normal emission with the X-ray source mounted at 54.44° with respect to the analyzer. Spectra were calibrated by rescaling the binding energy value to the Au 4f_7/2_ peak at 84 eV. Fitting analysis was performed using CasaXPS software introducing mixed Gaussian and Lorentzian contributions for each component. The background was fitted using the Shirley or linear method. The bulk reference sample was prepared by dropcasting a 2 mM solution of molecules. The semiquantitative elemental analysis was performed by employing the cross-section values extracted from the literature and removing the contribution of atomic sulfur present on surface.

### STM Measurements

The STM measurements were carried out by an Omicron Variable-Temperature VT-SPM setup operated in a vacuum using a Pt/Ir mechanically prepared tip. STM images of clean substrates were collected at room temperature, while samples with molecular monolayers were measured at 30 K, to stabilize the molecules during the scanning.

### Computational Methods

CP2K software was used for all pDFT calculations.^60^ RevPBE density functional,^61,62^ along with rVV10 nonlocal empirical dispersion corrections,^63^ were employed. Norm-conserving Goedecker-Tetter-Hutter pseudopotentials^64^ and a double-ζ basis set with polarization functions (DZVP-MOLOPT-SR) were used for all the atoms. The cell parameters were kept fixed throughout the optimizations, while all the atomic coordinates were let relax except the bottom layer of three slabs mimicking the Au(111) surface. The plane-wave cutoff value was set to 400 Ry. The wave function convergence (EPS_SCF) was set to 1.0 × 10^–6^, while the max force for the geometry optimization was set to 3 × 10^–4^ bohr^–1^ hartree. Orthorhombic cells of dimensions 17.31 Å × 40.39 Å × 40 and 26 Å × 25 Å × 40 were used for the simulation of a single chiral adsorbed molecule and for the monolayer, respectively.

### Substrate Preparation for mc-AFM Experiment

These substrates were prepared using an e-beam evaporation depositing on a clean Si wafer an adlayer of Ti (10 nm) followed by Ni (100 nm) and Au (10 nm). The substrate was then cleaned by immersing it in boiling acetone for 10 min and in boiling ethanol for additional 10 min. Finally, the substrate was kept under UV/ozone atm for 15 min.

### Self-Assembled Monolayer Deposition

The molecular monolayer was prepared by incubating a precleaned substrate in a 2 mM solution of **(***M***)-HelSAc** or **(***P***)-HelSAc** in dichloromethane overnight at room temperature. The surface was then rinsed several times with pure dichloromethane and dried under a N_2_ atmosphere.

### mc-AFM Measurements

The mc-AFM experiment was performed using a multimode magnetic scanning probe microscopy (SPM) system equipped with a Beetle-type Ambient AFM setup and an electromagnet with R9 electronic controller (RHK Technology). *I–V* measurements were performed under ±0.5 T magnetic field perpendicular to the sample surface at room temperature applying voltage ramps between ±0.3 V with a Pt-coated tip (DPE-XSC11, μmasch) in contact mode (applied force ca. 8–10 nN). At least 150 curves were scanned for each point, and several points were investigated all over the surface for a proper statistical analysis.

### MR Device Fabrication

Devices were fabricated by optical lithography, followed by e-beam evaporation. On a precleaned Si wafer 2 μm-wide Ti adlayer (8 nm) and Au (60 nm) were deposited by evaporation. The substrate was then cleaned by immersing it in boiling acetone for 10 min and in boiling ethanol for additional 10 min. Finally, the substrate was kept under UV/ozone atm for 15 min. On top of the gold layer, a self-assembled monolayer of molecules was deposited following the procedure previously described. Finally, as the top electrode, the insulating buffer layer of MgO (2 nm), Ni (40 nm), and Au (20 nm) layers were evaporated using a shadow mask with a line width of 20 μm. The device was subsequently attached to a cryogenic chip carrier and electrically connected by a wire bonder (Au wire). All electrical measurements were carried out within the cryogenics system made by Cryogenics, Ltd. A magnetic field of up to 1 T was applied perpendicular to the sample plane, and the resistance of the device was measured using the standard four-probe method. A constant current of 0.5 mA was applied using a Keithley current source (model 2400), and the voltage across the junction was measured using a Keithley nanovoltmeter (model 2182A).

## Supporting Information Available

Details on the synthesis procedure of **2** and **HelSAc**. ^1^H- and ^13^C NMR spectra of **2** and **HelSAc** (Figure S1 and S2). HPLC enantiomer resolution (Figure S3). ESI-MS characterization of **2** and **HelSAc** (Figure S4). N1s and C1s XPS regions of the bulk sample and of **HelSAc** monolayer assembled on gold (Figure S5 and S6). Semiquantitative XPS analysis of the bulk sample and of HelSAc monolayer assembled on gold (Table S1). STM image of bare annealed Au/mica substrate and diameter statistical analysis on the molecular monolayer (Figure S7). Nanoscratched AFM image and estimation of the molecular monolayer thickness (Figure S8). Data dispersion of a complete data set of *I–V* curves acquired in a single point of the surface (Figure S9) (PDF)
