# Supplementary material for: Efficient Spin-Selective Electron Transport at Low Voltages of Thia-Bridged Triarylamine Hetero[4]helicenes Chemisorbed Monolayer
Source: ACS Nano. 2023 Jul 26;17(15):15189–98. doi: 10.1021/acsnano.3c04878 (PMC10416567; doi:10.1021/acsnano.3c04878)
Supplement: Supplementary file 1 — nn3c04878_si_001.pdf [file nn3c04878_si_001.pdf]

Supplementary Information

Efficient spin-selective electron transport at low voltages of thia-bridged triarylamine hetero[4]helicenes chemisorbed monolayer.

*Niccolò Giaconi,‡ Lorenzo Poggini,† Michela Lupi, ‡ Matteo Briganti, ‡ Anil Kumar,§ Tapan K. Das,§ Andrea L. Sorrentino, ‡ Caterina Viglianisi, ‡ Stefano Menichetti, ‡ Ron Naaman,§ Roberta Sessoli, ‡ Matteo Mannini. ‡,**

‡ Department of Chemistry "Ugo Schiff" (DICUS) & INSTM Research Unit, University of Florence, Via della Lastruccia 3-13, Sesto Fiorentino, 50019, Italy. e-mail: matteo.mannini@unifi.it

† Istituto di Chimica dei Composti Organo-Metallici (ICCOM-CNR), Via Madonna del Piano 10, Sesto Fiorentino, 50019, Italy.

§ Department of Chemical and Biological Physics, Weizmann Institute of Science, Rehovot, 76100,

Israel.

Synthesis and characterization

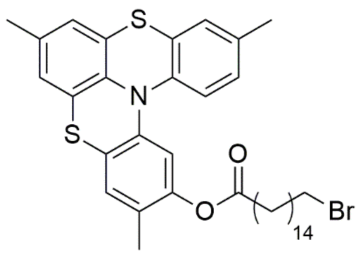

(±)-3,7,11-trimethyl[1,4]benzothiazino[2,3,4-kl]phenothiazin-2-yl 16-bromohexadecanoate (**2**): a round-bottom flask was charged with: helicene **1** (483 mg, 1.33 mmol), 16-bromohexadecanoic acid (577 mg, 1.73 mmol) and 4-(dimethylamino)pyridine (16 mg, 0.13 mmol). The solids were dissolved in 27 mL of dry CH_2Cl_2 and *N,N'*-diisopropylcarbodiimide (126 mg, 1.73 mmol) was added *via* syringe. The solution is stirred overnight at room temperature under a nitrogen atmosphere. The suspension was diluted with 160 mL of ethyl acetate, washed with a saturated solution of $NaHCO_3$ (60 mL × 2) and water (60 mL). The organic layer was dried over Na_2SO_4, filtered and the volatiles were eliminated under reduced pressure to afford 1,0 g of crude material. Purification by flash chromatography on silica gel (petroleum ether/CH_2Cl_2 -2:1, Rf: 0.50) gave the product as a white solid (830 mg, 92%). **mp** 74-76 °C. **IR (ATR solid)** ***ν*** = 2920 (C-H), 2851 (C-H), 1748 (C=O), 1490 (C=C), 1450 (C=C), 1313 (C-O), 1133 (C-O) cm. **Anal. calcd for $C_{37}H_{46}BrNO_2S_2$**: C, 65.28; H, 6.81; N, 2.06; S, 9.42. Found: C, 65.27; H, 6.86; N, 2.06; S, 9.47.**ESI-MS *m*/*z* (%):** 702 (67), 704 (77) $[M + Na]^+$, 1382 (100) $[2M + Na]^+$. **^1H NMR (400 MHz, CD_2Cl_2, δ)**: 7.06-7.09 (m, 2H; Ar H), 7.03 (bs, 1H; Ar H), 6.96 (dd, 1H *J*=8.2 Hz, *J*=2.0 Hz; Ar H) ppm 6.80-6.81 (m, 3H; Ar H), 3.43 (t, 2H, *J*=6.9 Hz, CH_2), 2.51 (t, 2H, *J*=7.6 Hz; CH_2), 2.29 (s, 3H; CH_3), 2.23 (s, 3H; CH_3), 2.11 (s, 3H, CH_3), 1.86 (qui, 2H, *J*= 7.4 Hz; CH_2), 1.70 (qui, 2H, *J*=7.3 Hz; CH_2), 1.28-1.45 (m, 22H; 11×CH_2), ppm. **^{13}C NMR (100 MHz, CD_2Cl_2, δ):**172.2, 149.4, 142.0, 140.1, 137.4, 135.4, 135.2, 129.8, 128.8, 128.6, 127.2, 127.1, 126.6, 126.5, 125.8, 125.8, 124.2, 120.7, 114.4, 34.8, 34.6, 33.5, 30.21, 30.20 (2C), 30.15, 30.13, 30.03, 30.01, 29.8, 29.7, 29.4, 28.8, 25.5, 20.9, 20.7, 16.0, ppm.

***(±)*-3,7,11-trimethyl[1,4]benzothiazino[2,3,4-kl]phenothiazin-2-yl 16-(acetylthio)hexadecanoate (HelSAc)**: to a suspension of K_2CO_3 (73 mg, 0.53 mmol) in freshly distilled THF, thioacetic acid was added (40 mg, 0.53 mmol). The mixture was stirred for 30' then helicene (300 mg, 0.44 mmol) was added and the reaction was stirred at room temperature for 30 h under a nitrogen atmosphere. The reaction was diluted with 80 mL of ethyl acetate and washed with water (30 mL × 2). The organic layer was dried over Na_2SO_4, filtered and the volatiles were eliminated under reduced pressure to afford 280 mg of crude material. Purification by flash chromatography on silica gel (petroleum ether/CH_2Cl_2 -3:2, Rf: 0.46) gave the product as a white solid (230 mg, 83%).

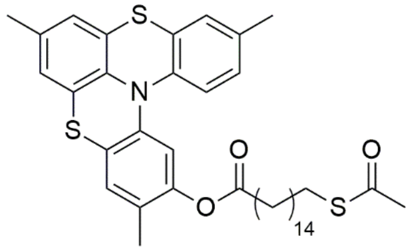

mp 72-74 °C. **IR (ATR solid) *ν* =** 2922 (C-H), 2852 (C-H), 1757 (C=O), 1690 (C=O), 1486 (C=C), 1449 (C=C), 1133 cm (C-O)$^{-1}$ **Anal. calcd for $C_{39}H_{49}NO_3S_3$**: C, 69.29; H, 7.31; N, 2.07; S, 14.23. Found: C, 69.26; H, 7.33; N, 2.10; S, 14.25. **ESI-MS *m*/*z* (%):** 698 (59) $[M+Na]^+$ 1372 (100)$[2M+Na]^+$ **UV-Vis** (*CH_2Cl_2*): λ_{max} (ε) = 314 (9172). **^{1}H NMR (400 MHz, CD_2Cl_2, δ)**: 7.10 (d, 1H, *J*=8.2 Hz; Ar H), 7.02 (s, 1H; Ar H), 6.99 (bs, 1H; Ar H), 6.93 (dd, 1H, *J*=1.8 Hz, *J*=8.2 Hz; Ar H), 6.81 (s, 1H; Ar H), 6.76-6.77 (m, 2H, Ar H), 2.86 (t, 2H, *J*=7.4Hz, CH_2), 2.49 (t, 2H, *J*=7.6 Hz, CH_2), 2.32 (s, 3H, CH_3), 2.27 (s, 3H, CH_3) 2.20 (s, 3H, CH_3), 2.09 (s, 3H, CH_3), 1.71 (qui, 2H, *J*=7.6Hz; CH_2), 1.56 (qui, 2H, *J*=7.1 Hz; CH_2), 1.25-1.36 (m, 22H; 11×CH_2), ppm. **^{13}C NMR (100 MHz, CD_2Cl_2, δ):** 196.2, 171.9, 148.8, 141.6, 139.8, 137.0, 134.7, 134.4, 129.4, 128.4, 128.2, 126.7, 126.4, 126.2, 126.1, 125.44, 125.37, 123.9, 120.3, 114.1, 34.3, 30.8, 29.76 (2C), 29.75, 29.70 (2C), 29.64, 29.61, 29.56, 29.4, 29.30 (2C), 29.26, 29.0, 25.1, 20.7, 20.5, 15.8, ppm.

^{1}H and ^{13}C NMR

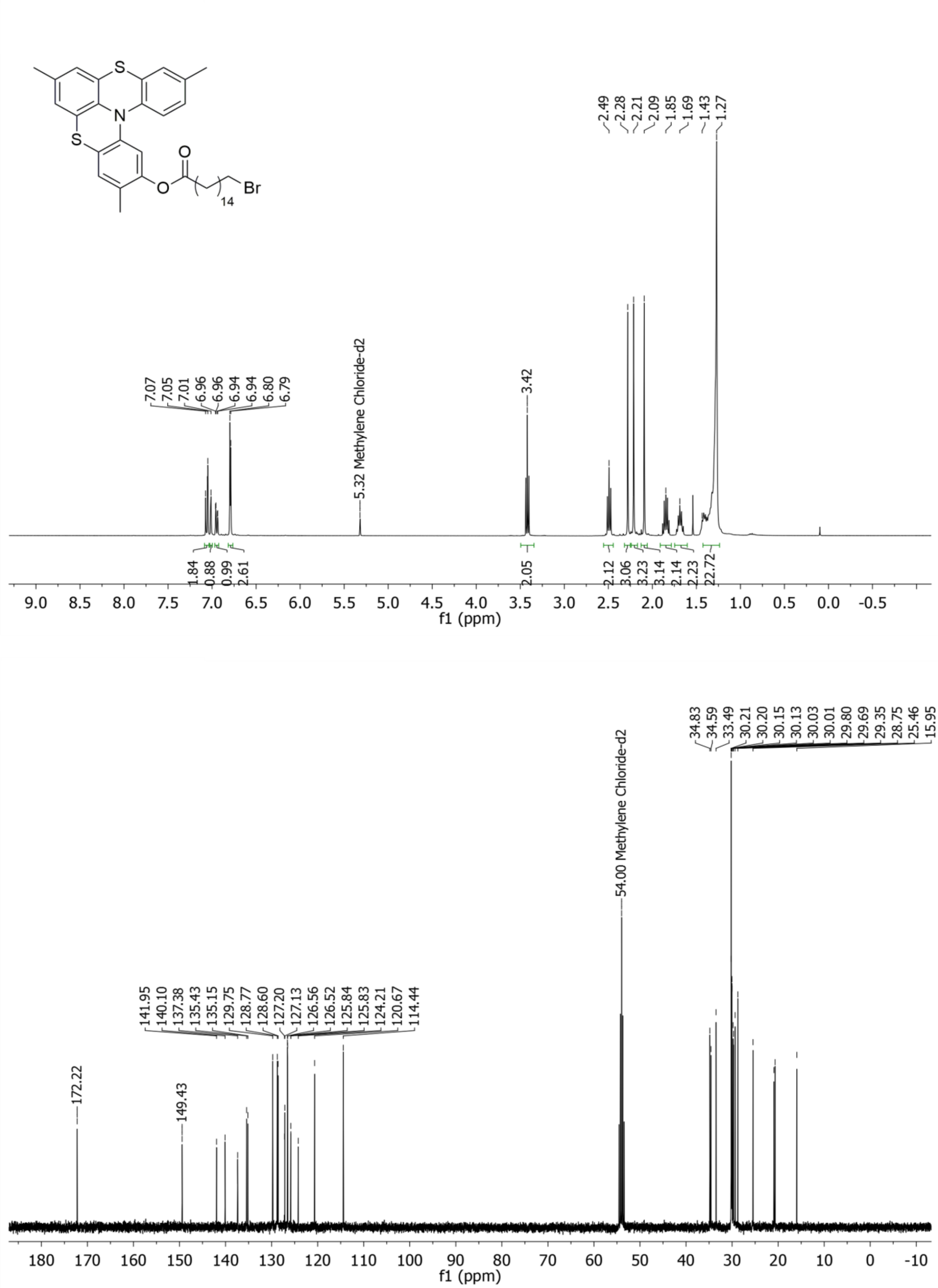

Figure S1*:* ^{1}H and ^{13}C NMR spectra of **2**

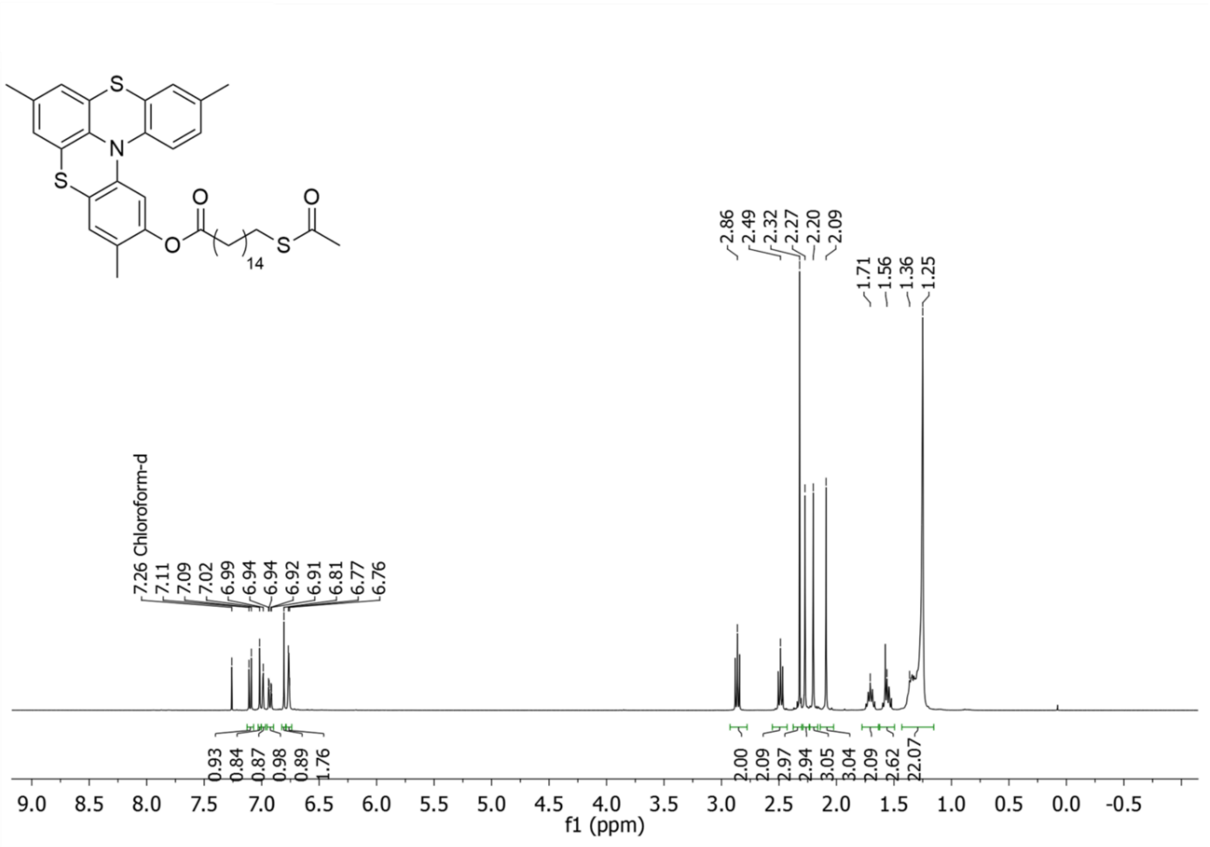

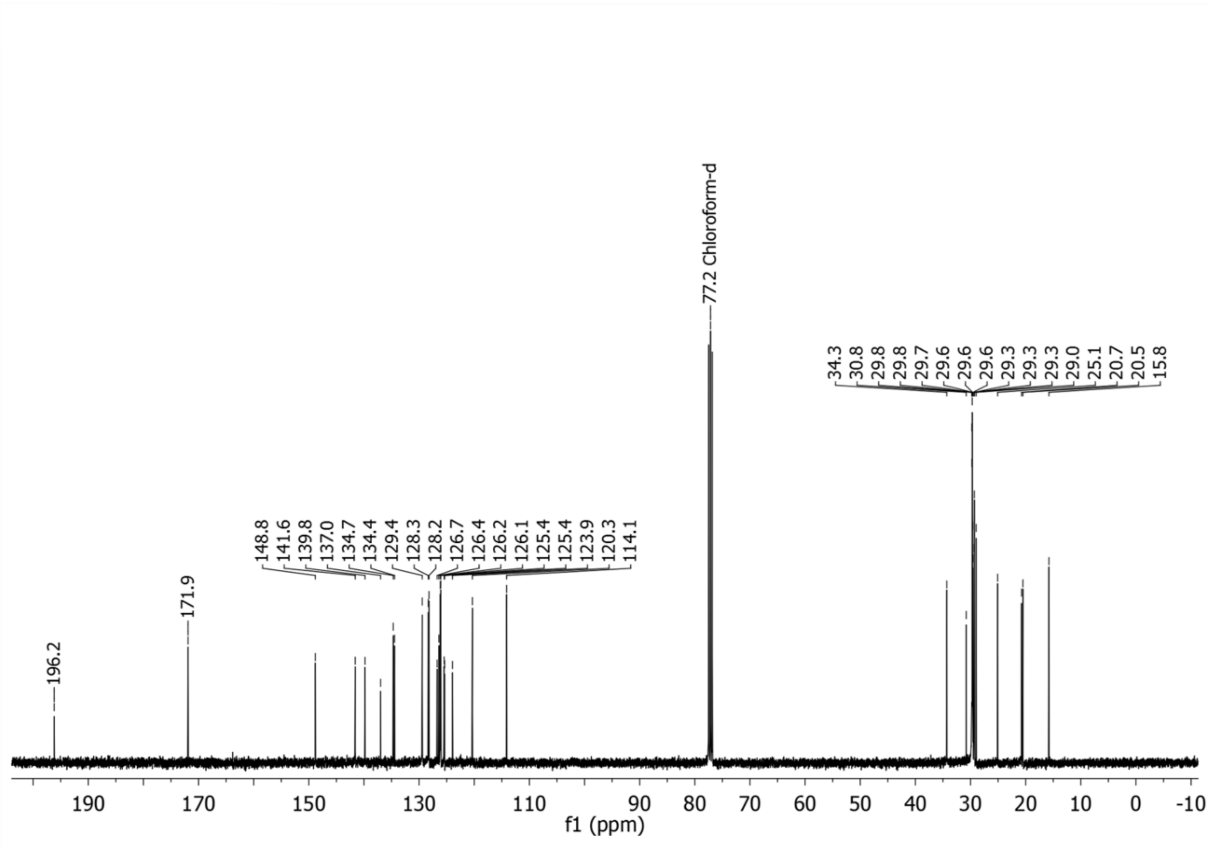

Figure S2: ^{1}H and ^{13}C NMR spectra of **HelSAc**

HPLC enantiomer resolution

HelSAc

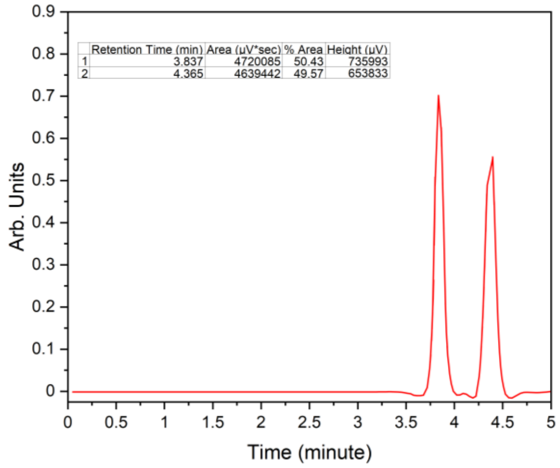

(*P*)-HelSAc

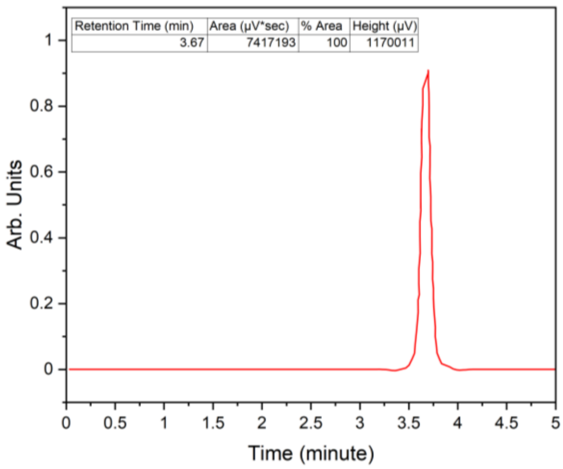

(*M*)-HelSAc

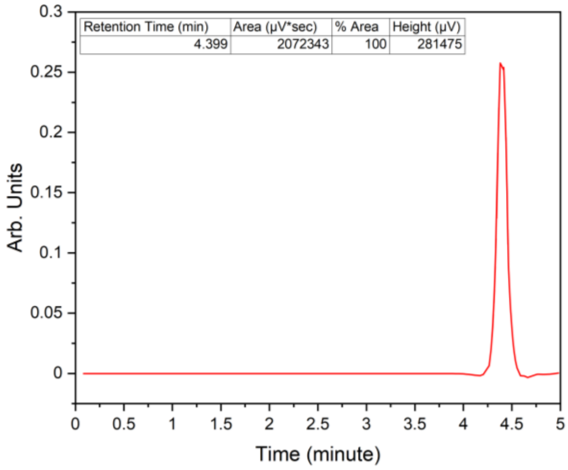

Figure S3*:* Analytical HPLC analysis after semipreparative HPLC resolution of **HelSAc**

ESI-MS

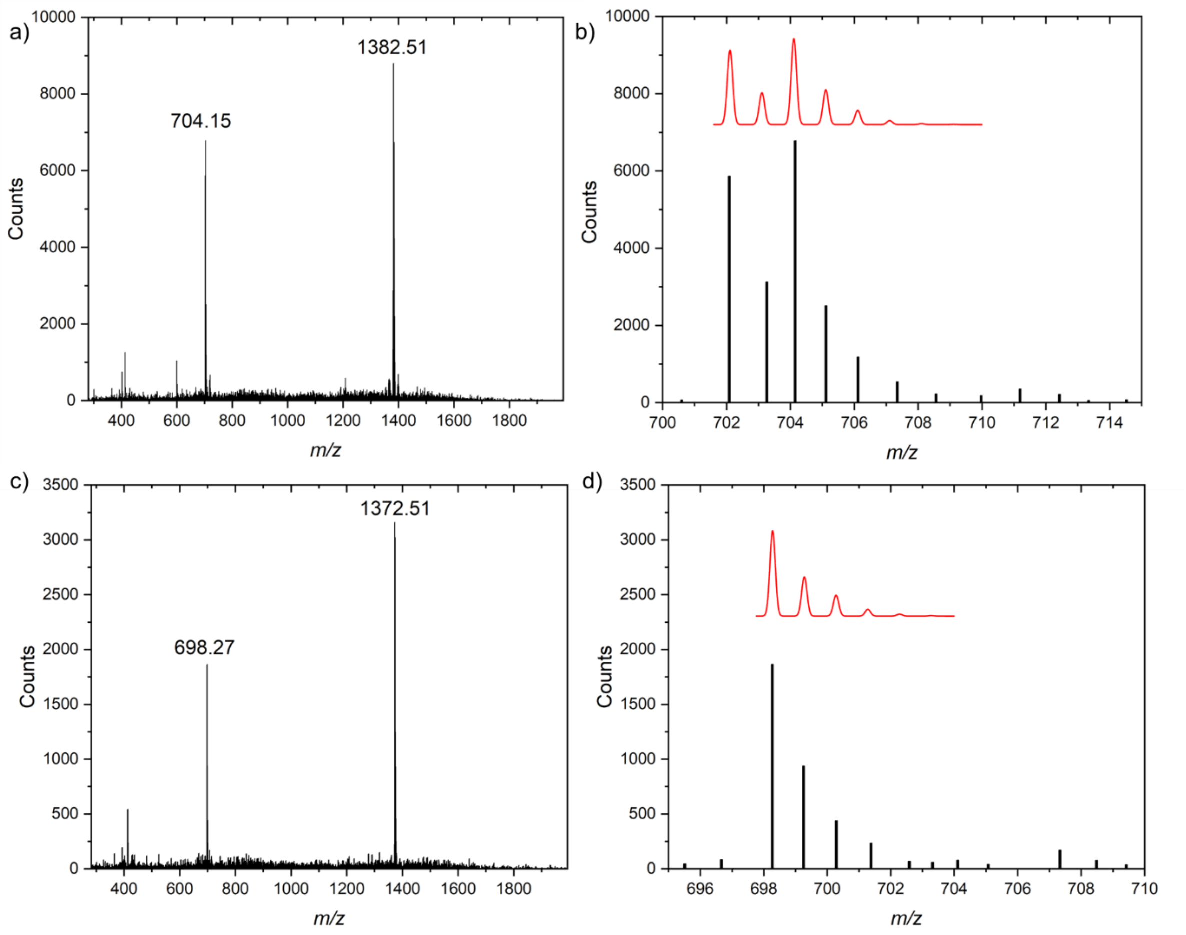

Figure S4: ESI-MS characterization of **2** (a-b) and **HelSAc** (c-d). Red lines above the experimental data represent the theoretical isotopic distribution of the most significant signals.

XPS characterization

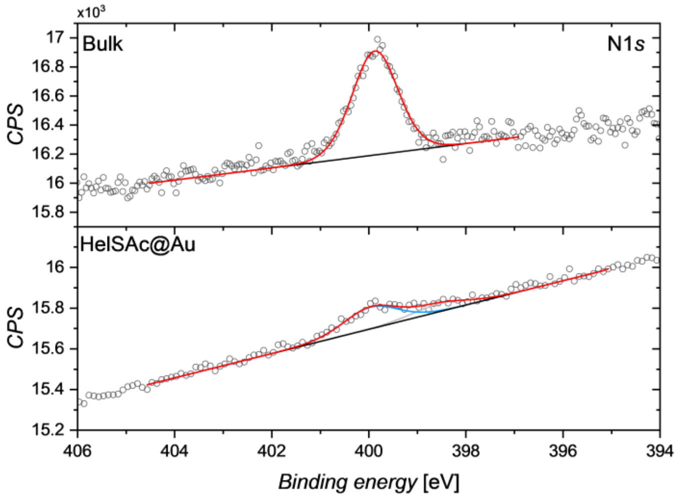

Figure S5*:* N1*s* XPS region of **HelSAc** bulk sample (top) and of **HelSAc@Au** (bottom).

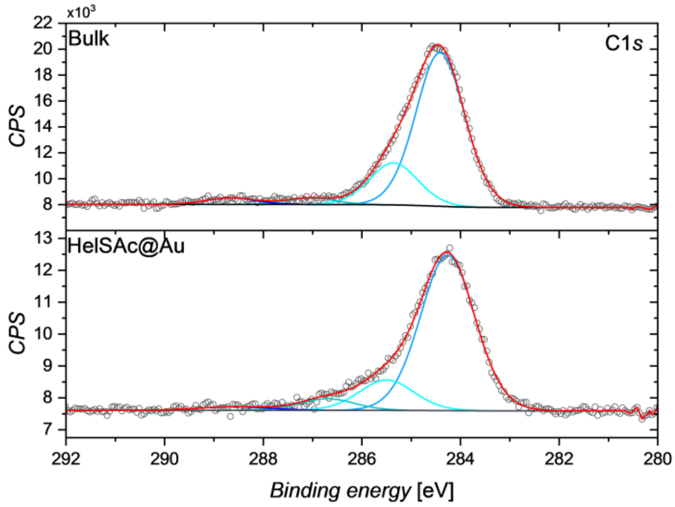

Figure S6*:* C1*s* XPS region of **HelSAc** bulk sample (top) and of **HelSAc@Au** (bottom).

Sample	S2*p*[%]	N1*s*[%]	C1*s*[%]	S/N ratio
Expected bulk composition	7.1	2.4	90.5	3
Bulk	6.5±0.3	2.2±0.1	91.4±4.6	3
Expected SAM composition	75	25	--[b]	3
HelSAc@Au	74.5±3.7[a]	25.5±1.3	--[b]	2.9

[a] The contribution from atomic sulfur at 161.3 eV has been excluded to properly evaluate the stoichiometry of the chemisorbed material on top of the gold substrate. [b] The contribution of carbon for semi-quantitative analysis of SAM has been excluded due to the presence of environmental contamination.

Table S1*:* XPS semi-quantitative analysis of **HelSAc** bulk sample and of **HelSAc@Au** .

STM characterization

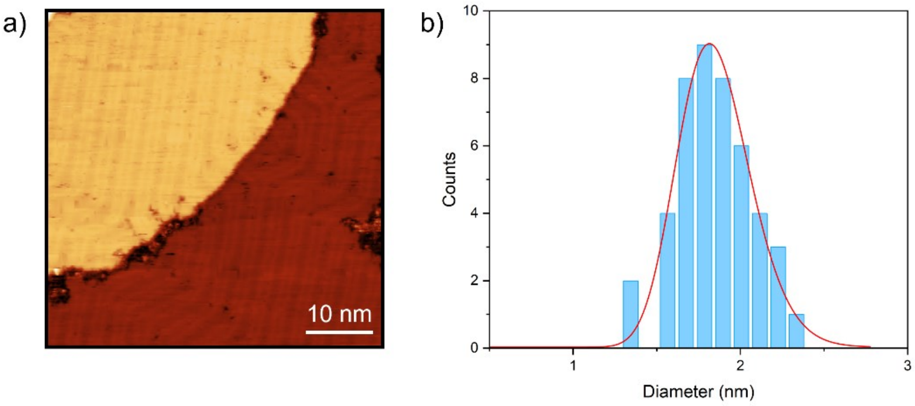

Figure S7: a) STM image of bare annealed Au/mica substrate recorded at room temperature (50x50 nm^2, V=2 V, I_t=200 pA). b) Statistical analysis extracted from the **Figure 1e**.

mc-AFM characterization

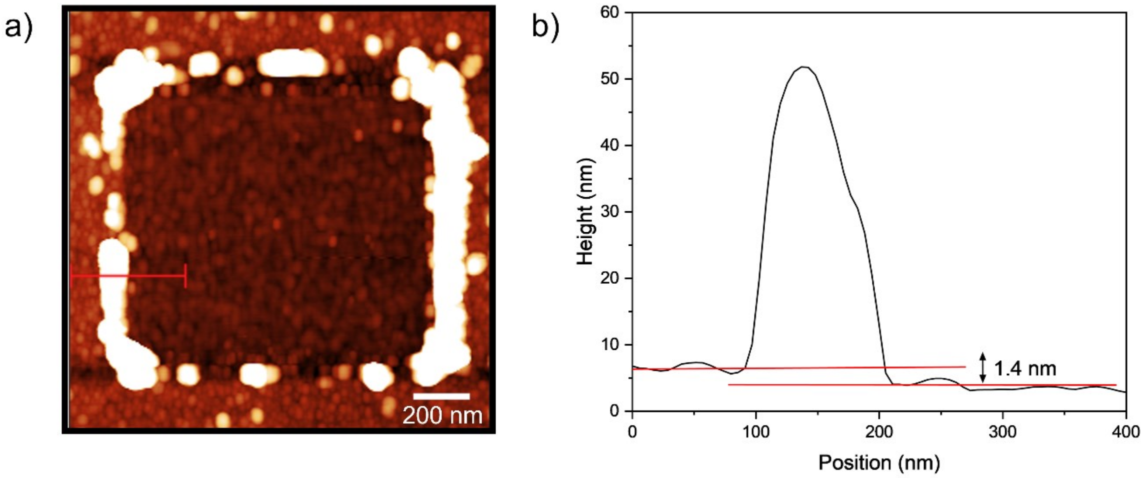

Figure S8: a) AFM nano-scratched image of ***(M)*-HelSAc@Au**. b) Height profile extracted from a) evidencing an estimation of 1.4 nm thickness of the molecular deposit.

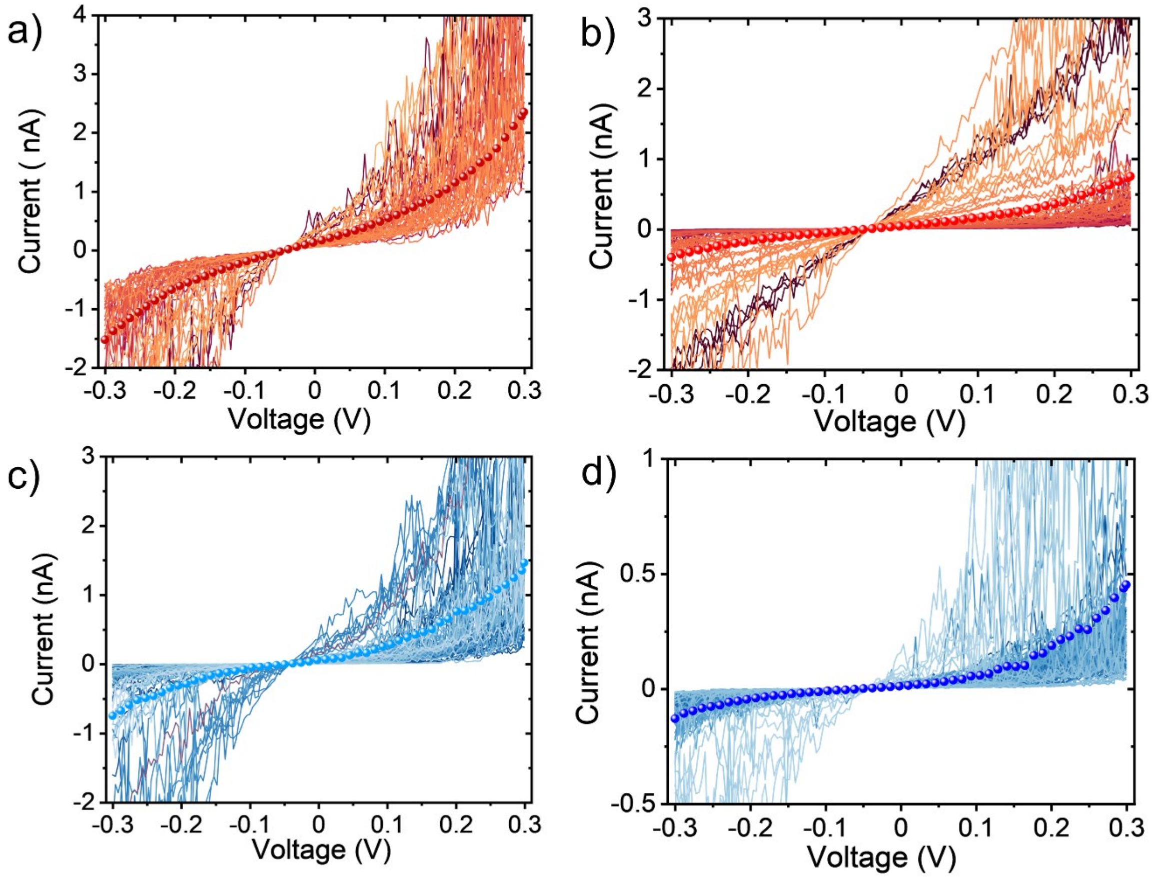

Figure S9: Complete dataset of mc-AFM measurements acquired on (*M*)-HelSAc (red) and on (*P*)-HelSAc (blue) by applying either a-d) positive or b-c) negative magnetic field.
